# Using data science to investigate rising HIV low-level viraemia results at Groote Schuur laboratory in South Africa

**DOI:** 10.4102/ajlm.v14i1.2953

**Published:** 2025-12-20

**Authors:** Mo Se Kwon, Khumo O. Sematle, Lucia Hans, Stephen Korsman, Nei-yuan Hsiao, Diana R. Hardie

**Affiliations:** 1 Division of Medical Virology, Department of Pathology, Faculty of Health Sciences, University of Cape Town, Cape Town, South Africa; 2 National Health Laboratory Service, Cape Town, South Africa; 3 Department of Molecular Medicine and Haematology, Faculty of Health Sciences, University of the Witwatersrand, Johannesburg, South Africa; 4 National Priority Programme, National Health Laboratory Service, Johannesburg, South Africa

**Keywords:** laboratory testing, HIV, viral load, data science, low-level viraemia, pre-analytical factors, ART programme, contamination

## Abstract

**Background:**

Following a major service disruption across the National Health Laboratory Service, backlogged HIV viral load (VL) specimens from Limpopo province were rerouted to Groote Schuur Hospital (GSH) laboratory in the Western Cape province. During this time, an increase in low-level viraemia (LLV; 50 copies/mL – 1000 copies/mL) was observed at the GSH laboratory, raising concerns about possible pre-analytical and analytical issues, including compromised specimen quality and possible contamination.

**Objective:**

To determine whether the observed increased LLV was due to analytical errors (e.g. contamination), pre-analytical factors such as prolonged turnaround times (TAT), or underlying epidemiological differences.

**Methods:**

HIV VL data from 2023–2024 for Limpopo and the Western Cape were analysed using Python. Viral load results were grouped into predefined categories and compared. Turnaround times were plotted and Thembisa model estimates were used to assess HIV prevalence and antiretroviral therapy (ART) coverage. Longitudinal patient-level analysis evaluated VL trends as a proxy for adherence. In addition, quality control data were evaluated at testing sites.

**Results:**

Limpopo specimens showed higher LLV (20%) and lower viral suppression (< 50 copies/mL) at 70%, compared to Western Cape (13% LLV, 81% suppression), where follow-up and suppression outcomes were also higher. No clear evidence indicated that extended TAT or potential instrument contamination affected VL results significantly.

**Conclusion:**

The increase in LLV at GSH was linked primarily to processing specimens from Limpopo, highlighting regional differences in HIV VL result distributions. These differences probably reflect variations in ART access and adherence, rather than laboratory-related issues such as delayed TAT, sample quality, or contamination.

**What this study adds:**

This study shows how province-specific HIV result patterns correlate with ART adherence, using a Python script to assess serial VLs and follow-up suppression. It demonstrates the value of routine data analysis in monitoring high-throughput HIV VL tests, which are often auto-released without pathologist oversight.

## Introduction

Viral load (VL) testing is the cornerstone of monitoring HIV-1 viraemia in people living with HIV who have been initiated on antiretroviral therapy (ART). Viral load testing does not only monitor treatment success but also informs clinical decisions regarding adherence interventions and regimen changes in cases of virologic failure. The National Health Laboratory Service (NHLS) in South Africa provides HIV VL testing at 17 centralised laboratories across the country,^1^ with smaller local laboratories referring samples to these reference facilities.

In October 2024, during an internal quality assessment at the Groote Schuur Hospital (GSH) laboratory, we noted an increase in low-level viraemia (LLV) results, defined as HIV VLs between 50 copies/mL and 1000 copies/mL, as well as a rise in values below the limit of quantification. The number of results reported as lower than detectable (LDL) decreased, while HIV VLs > 1000 copies/mL remained stable.

The mechanisms underlying LLV remain poorly understood. Patients with LLV are often maintained on failing regimens, which can promote the accumulation of drug-resistant mutants, virologic rebound, increased risk of virological failure, poorer clinical outcomes, and ongoing transmission.^2^ It is essential to distinguish sustained LLV from intermittent LLV, also known as ‘blips’, which are typically short-lived and carry a lower risk of resistance or disease progression.

The recent rise in LLV rates in South Africa is particularly concerning, especially following the nationwide rollout of dolutegravir by 2022, which was expected to enhance viral suppression.^3^ This trend mirrors observations from other Southern African Development Community countries, such as Botswana,^4^ where dolutegravir scale-up has similarly not led to the anticipated reductions in virologic failure and LLV prevalence.

Upon further enquiry, it was noted that the GSH laboratory had started receiving a large volume of specimens from another central laboratory in the Limpopo province, outside of the usual catchment area of our laboratory, citing operational difficulties and testing backlogs. It is worth noting that most HIV samples in Limpopo are processed at one centralised laboratory in the province, located approximately 1739 kilometres from GSH, which is located in the Western Cape province.^5^ The transit times for these specimens were inevitably prolonged as they were transported by road to GSH, and the specific conditions under which specimens were transported could not be confirmed (i.e. to what degree the cold chain was maintained).

This study aims to evaluate HIV VL data from both laboratories and to investigate whether the increase in LLV observed at GSH is associated with an increase in referred specimens from outside the province, and whether the extended transit times affected sample quality and VL results. An accompanying increase in reactive negative controls also raised concerns regarding possible contamination and false positive results. The study further considers whether the current national virological failure threshold (> 1000 copies/mL) remains appropriate, and explores how ART coverage and adherence may influence the distribution of VL results across provinces.

## Methods

### Ethical considerations

This study was conducted in accordance with the ethical principles outlined in the Declaration of Helsinki. Ethical approval was granted by the Human Research Ethics Committee of the University of Cape Town (reference number: 233/2025). Approval to access and analyse data from the NHLS database was obtained through the NHLS Academic Affairs and Research Management System.

Informed consent was waived, as all published data is aggregated and contains no personal identifiers. Data were analysed and stored on a password-locked computer and only accessible to the study co-investigators.

### HIV viral load testing

HIV-1 VL testing was performed using the Abbott Alinity m HIV-1 assay (Abbott Molecular, Des Plaines, Illinois, United States), a fully automated real-time, reverse transcriptase polymerase chain reaction system used in South African laboratories affiliated with the NHLS. Prior to testing, specimen tubes were centrifuged at 3000 g for 10 min to separate plasma or serum. The assay processes 0.2 mL to 1.0 mL of plasma or serum, extracting HIV-1 RNA via magnetic microparticle technology and amplifying two highly conserved genomic regions alongside an internal RNA control to monitor assay performance. For specimens with volumes less than 0.2 mL, a 1:5 dilution is prepared and analysed using the 1 mL protocol. The assay demonstrates a limit of detection of 20 copies/mL and a quantification range of 20 copies/mL – 10 000 000 copies/mL, with the lower limit of quantification dependent on input volume (50 copies/mL for 0.2 mL protocol; 20 copies/mL for 1.0 mL protocol). It is validated for HIV-1 groups M (subtypes A–H and circulating recombinant forms), O, and N, ensuring broad subtype inclusivity for accurate clinical monitoring.^6^

### Data collection

Access to patient-level HIV VL data stored in the NHLS Corporate Data Warehouse in Johannesburg was obtained through the Academic Affairs and Research Management System. Following institutional approval, HIV VL data from the TrakCare laboratory information system were extracted for the Limpopo and Western Cape provinces, covering the period from 2023 to 2024. The data were provided in comma-separated values (.csv) format. To investigate the potential impact of laboratory contamination on negative control results, relevant maintenance records were requested from an Abbott representative for both laboratories.

In addition, contextual information on HIV prevalence and ART coverage was sourced from the Thembisa model^7^ — a comprehensive, publicly available HIV modelling tool developed for South Africa. The model integrates demographic, behavioural and treatment-related data to estimate HIV trends and assess intervention impacts, and it is updated routinely at both national and provincial levels.

### Data/statistical analysis

Data were analysed using a custom Python script developed with the pandas library (Python Software Foundation, Wilmington, Delaware, United States), an open-source data analysis toolkit used widely in data science for efficient manipulation, cleaning and transformation of structured data.

There were two main datasets: one extracted for Limpopo province and the other for the Western Cape province. The initial step of the script involved a data clean-up process, during which duplicate records, records falling outside the specified time period, entries with incorrect data types or formats, and records with missing information in key fields were removed. After the clean-up process, the number of HIV VL records from the Western Cape between 01 January 2023 and 31 December 2024 was 818 989, while the Limpopo dataset contained 1 169 903 records. The script then aggregated individual VL results into predefined ranges: LDL, < 50 copies/mL, 50–199 copies/mL, 200–1000 copies/mL, and > 1000 copies/mL. Results below the limit of quantification (< 20 copies/mL or < 50 copies/mL depending on volume protocol), as well as absolute values < 50 copies/mL, were grouped into the ‘< 50’ category. Monthly counts were grouped based on specimen ‘Collection date’ for both laboratories (Online Supplementary Tables 1–5).

For comparative purposes, absolute monthly counts were converted into percentages of total VL tests per month. These data were visualised using clustered bar graphs in Microsoft Excel (Microsoft Corporation, Redmond, Washington, United States) to facilitate interpretation (Online Supplementary Figures 1–4). Monthly turnaround time (TAT) was plotted on the same graph as a secondary line chart, measured in days. Turnaround time was calculated as the difference between ‘Registration Date’ (when specimens were logged into the laboratory information system) and ‘Entered Date’ (when test results were entered automatically post-processing on the Alinity m platform).

A separate chart was created to assess operations in Limpopo by grouping the number of specimens by month using the ‘Collection date’ field and again with the ‘Entered Date’ field. This helped to evaluate workflow efficiency, identify potential backlogs, and determine whether delayed processing had contributed to increased TATs and the referral of specimens to reference laboratories outside the province. If the number of specimens collected in a given month exceeded the number processed, it suggested the presence of a backlog.

To quantify objectively the deviations observed in VL distributions, 2024 monthly results were compared with corresponding 2023 data within each VL range. *Z*-scores were calculated as standardised deviations (*Z* = [result – mean]/standard deviation) based on 2023 means and standard deviations, allowing consistent comparison across categories (Online Supplementary Tables 1–5). Heatmaps generated using the Seaborn Python library visually represented these standardised deviations over time.

This study also examined potential epidemiological factors contributing to VL distribution changes.

Longitudinal patient-level VL data were analysed to assess ART adherence and virological suppression by examining serial VL results, follow-up testing rates, mean intervals between tests, and suppression rates. In the Western Cape, where a unique medical record number is assigned consistently to each patient, VL results were aggregated using this identifier. In contrast, for Limpopo and other provinces that do not use a standardised medical record number system, a probabilistic matching approach was used. A record linkage algorithm was developed to generate a synthetic ‘Unique_ID’, based on a weighted scoring system incorporating patient attributes such as name, surname, date of birth, sex, folder number, and hospital attended. Pairs of records exceeding a predefined similarity threshold were grouped as belonging to the same individual. Given the computational intensity of large-scale pairwise comparisons, records were first pre-clustered by the first letter of the surname to enhance processing efficiency. No patient identifiers are present in the output data and only aggregated results were analysed.

### Use of artificial intelligence

Artificial intelligence tools such as ChatGPT (OpenAI), Claude AI (Anthropic), and DeepSeek were used to assist in the development and refinement of the Python scripts used for data processing and analysis. These tools helped to correct syntax errors, troubleshoot logic issues, and optimise script functionality. In addition, they were used to improve grammar, clarity and consistency in the manuscript.

At no point was any patient-identifiable information or unprocessed data uploaded into any artificial intelligence platform. All analyses were conducted locally in full compliance with institutional data governance policies and ethical guidelines.

The use of artificial intelligence was limited to supportive roles in coding and editorial assistance. All data interpretation, analysis, and conclusions were independently conducted and verified by the authors.

## Results

### Groote Schuur Hospital laboratory

**FIGURE 1:**
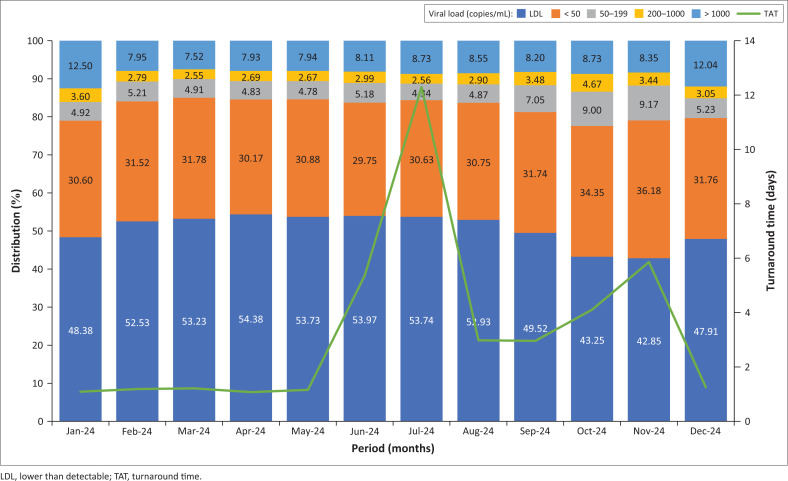
HIV viral load result distribution for Groote Schuur Hospital laboratory grouped monthly by ‘Collection date’ from January 2024 to December 2024 (*N* = 204 966, average of 17 081 specimens collected per month).

Between February 2024 and August 2024, the distribution of HIV VL results at GSH remained consistent. On average, 53% of results were classified as LDL, 31% were < 50 copies/mL, 4.9% fell within 50–199 copies/mL, 2.7% within 200–1000 copies/mL, and 8.1% were > 1000 copies/mL (Figure 1). Notable increases in VLs above 1000 copies/mL were seen in January 2024 and December 2024, probably reflecting a subset of newly diagnosed individuals who had not yet started treatment and were not yet engaged in routine ART clinic follow-up.

**FIGURE 2:**
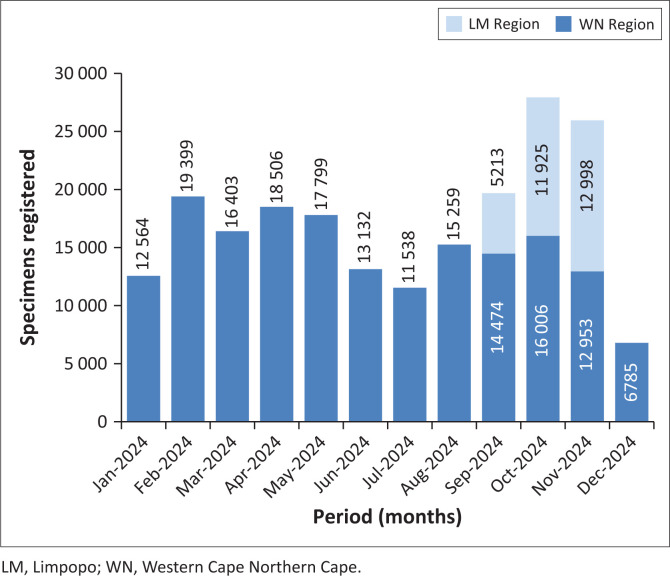
HIV viral load specimen volume at Groote Schuur Hospital laboratory grouped monthly by ‘Collection date’ and by ‘Episode region’, from January 2024 to December 2024.

**FIGURE 3:**
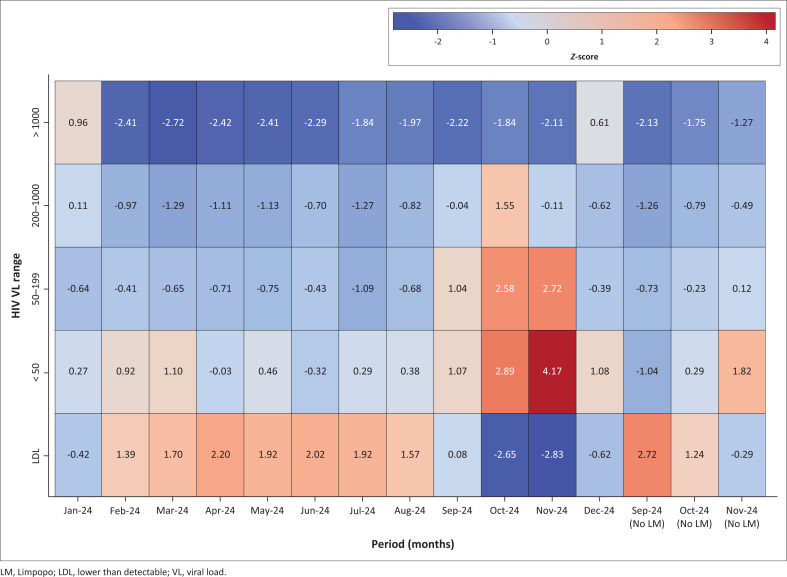
Heatmap illustrating how HIV viral load distribution at Groote Schuur Hospital from January 2024 to December 2024 deviated from its reference data.

From September 2024 onwards, a shift was observed in the VL distribution. Lower than detectable results began to decline, while the proportions in the < 50, 50–200, and 200–1000 categories increased (> 1000 copies/mL category remained unchanged). The most significant changes occurred in November 2024, with LDL dropping to 42.85%, < 50 rising to 36.18%, and 50–199 increasing to 9.17%. Figure 3 shows that these changes exceeded two standard deviations, especially in October 2024 and November 2024. These months coincided with a surge in sample referrals from Limpopo: 5213 in September 2024, 11 925 in October 2024, and 12 998 in November 2024 (Figure 2).

**FIGURE 4:**
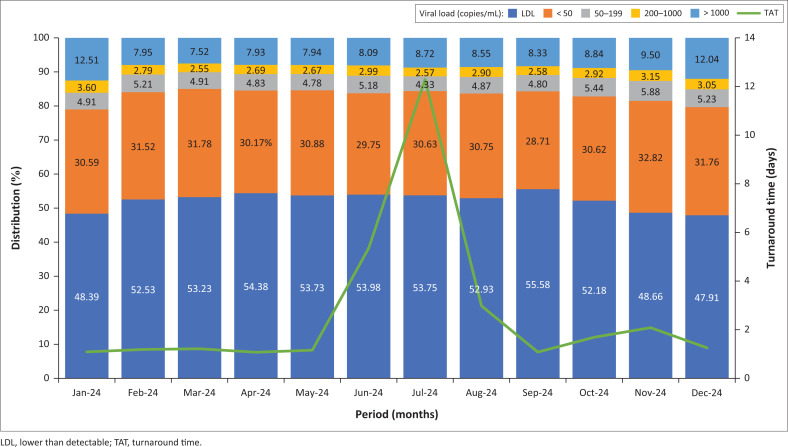
HIV viral load result distribution for Groote Schuur Hospital laboratory grouped monthly by ‘Collection date’ from January 2024 to December 2024 (excluding samples from the Limpopo region).

To determine whether this influx affected overall patterns, the analysis was repeated excluding Limpopo specimens. When only Western Cape (Western Cape Northern Cape catchment area) data were included, the VL distributions remained steady after August 2024, mirroring the earlier part of the year. *Z*-scores also remained mostly below 2 (Figure 3 and Figure 4), indicating no significant deviations. While TATs appeared longer in June 2024 and July 2024, as a result of retrospective data entry after the cyberattack, the actual VL distributions during this period remained stable (*Z*-score < 3), despite reduced specimen numbers (Figure 2).

### Contamination event in 2024

Groote Schuur Hospital laboratory observed an increase in reactive negative controls during November 2024 and December 2024, with seven reactive cases reported in November 2024 and six in December 2024, compared to only zero or one per month in the preceding months. There was also an increase in positive environmental controls, with VLs ranging between < 20 copies/mL up to log 3 copies. This increase coincided with a surge in testing volume driven by external referrals, which may have elevated the risk of contamination events. Compounding the situation, the Limpopo laboratory was concurrently managing its own contamination issue, evidenced by a spike of 13 reactive negative controls in November 2024 (average of 1.4 reactive negative controls in preceding months), which prompted a temporary shutdown of one of its instruments to carry out a full decontamination procedure.

No notable deviation in the HIV VL distribution was observed in the GSH data in 2024 after excluding results from Limpopo specimens during this period, as illustrated in the heatmap and clustered bar graph (Figure 3 and Figure 4). The contamination event was resolved following standard maintenance and decontamination procedures, including ethanol-based cleaning of the amplification area, with no further spikes in reactive negative controls observed in the couple of months that followed (Online Supplementary Table 6).

### Specimens collected in Limpopo province

**FIGURE 5:**
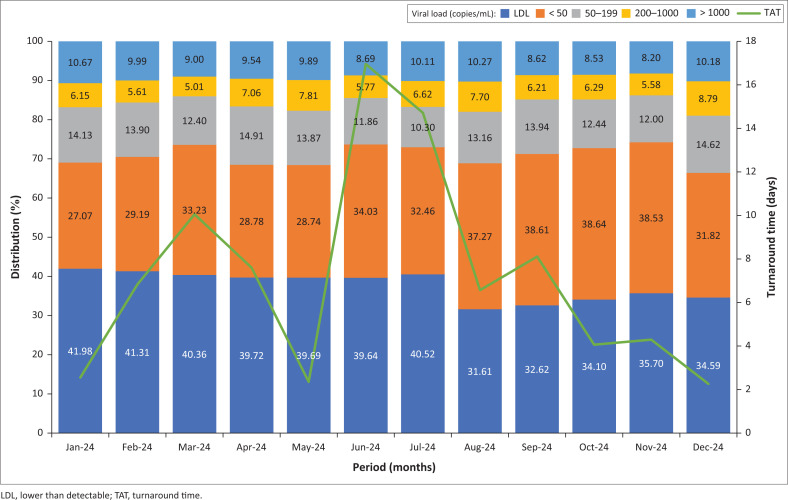
HIV viral load result distribution for Limpopo specimens grouped monthly by ‘Collection date’ from January 2024 to December 2024 (N = 614 840, average of 51 237 specimens collected per month).

Figure 5 shows the overall distribution of VL results for Limpopo specimens, grouped by VL range. From September 2024, some specimens were sent to external laboratories such as GSH owing to local backlogs, although most were still tested within Limpopo (Online Supplementary Figure 5). Interestingly, the VL distribution for Limpopo differed noticeably from those seen at GSH and across the Western Cape (Online Supplementary Figures 1–4).

Between January 2024 and July 2024, Limpopo’s VL results were fairly stable. The < 50 copies/mL category showed minor fluctuations in March, June, and July 2024, averaging about 33% of results. On average, VL results during this period were distributed as follows: 41% LDL, 31% below 50 copies/mL, 13% between 50 and 199, 6.3% between 200 and1000, and 9.7% above 1000 copies/mL. From September 2024 onwards, the LDL proportion dropped to about 34%, while < 50 copies/mL increased to about 37%. The other categories remained largely unchanged (Online Supplementary Figure 6).

Turnaround times peaked at about 10 days in March 2024, probably because of high specimen volumes that overwhelmed local processing capacity. Another peak occurred during the June 2024 to July 2024 cyberattack period, although this was partly because of delays in retrospective data capture (Online Supplementary Figure 5). Despite these operational issues, VL distributions remained stable up to July 2024, with only slight increases in the < 50 category (*Z*-score > 3) in March, June, and July 2024 (Online Supplementary Figure 6).

**FIGURE 6:**
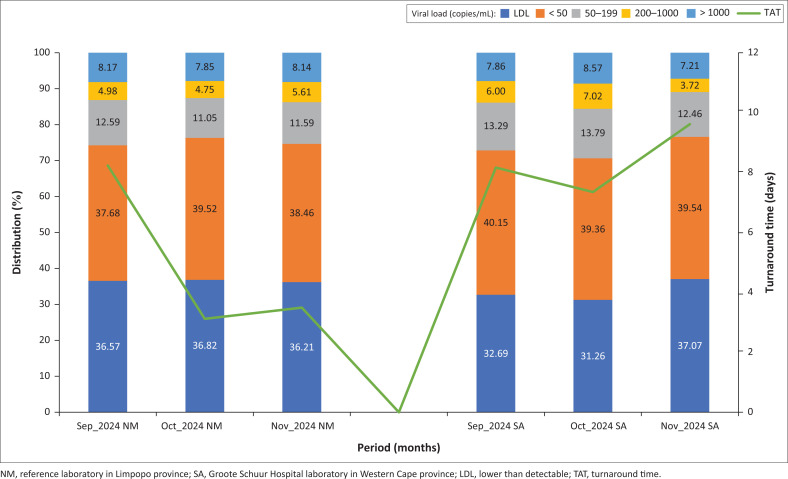
HIV viral load result distribution for Limpopo specimens by test site from September 2024 to November 2024.

As lab operations improved after August 2024, TAT decreased steadily, reaching just two days by December 2024. Strangely, this coincided with a drop in LDL results and a rise in the < 50 copies/mL category. A comparison of results by testing site (Limpopo laboratory vs GSH) showed no major differences in VL patterns or any consistent link between TAT and VL distribution (Figure 6, Online Supplementary Figure 7, and Online Supplementary Figure 8).

### HIV prevalence, antiretroviral therapy coverage, and adherence data

According to the 2024 estimates of the Thembisa model, HIV prevalence in the Western Cape was approximately 6.9%, with ART coverage about 74%. In comparison, Limpopo had an estimated HIV prevalence of 9.8% and an ART coverage rate of 72.4%, of which the national HIV prevalence is 12.8% and ART coverage is 77.7%.^7^

By using a custom record linkage algorithm on Python, an analysis of the 2023–2024 Limpopo data identified 590 136 unique patients, excluding those with only one VL test conducted after 31 August 2024 (as these patients are most likely newly diagnosed and unlikely to have had follow-up VLs by the end of the dataset on 31 December 2024). Among these patients, 301 953 instances of VL ≥ 50 copies/mL were recorded, of which more than one instance can belong to a single patient. Of these, only 151 340 instances (50.12%) had a follow-up VL result, with a mean time to follow-up of 211 days. Among those with follow-up tests, 74 281 (49.08%) achieved virological suppression (< 50 copies/mL).

In the Western Cape, 402 537 unique patients were identified after applying the same exclusion criteria. There were 164 025 instances of VL ≥ 50 copies/mL, with 89 732 (54.71%) followed up. The average time to follow-up was 225 days, and 48 711 (54.28%) of those followed up were virologically suppressed (Table 1).

**TABLE 1:** Distribution of unique patients by initial HIV viral load category, with corresponding follow-up testing percentages, average number of days to follow-up, and follow-up viral load outcomes categorised by the same predefined ranges, in Limpopo and Western Cape provinces, 01 January 2023 to 31 December 2024.

Province and number of unique patients	Initial viral load (copies/mL)	Total episodes	Episodes with F/U	F/U (%)	Average days to F/U	F/U VL < 50 copies/mL		F/U VL 50–199 copies/mL		F/U VL 200–1000 copies/mL		F/U VL > 1000 copies/mL	
						*n*	%	*n*	%	*n*	%	*n*	%
Limpopo (*n* = 590 136)													
	< 50	448 763	207 661	46.27	297.1	159 672	76.89	34 523	16.62	8075	3.89	5391	2.60
	50–199	166 731	78 769	47.24	237.9	46 016	58.42	22 543	28.62	6013	7.63	4197	5.33
	200–1000	55 960	29 640	52.97	197.1	13 970	47.13	8329	28.10	3806	12.84	3535	11.93
	> 1000	79 262	42 931	54.16	170.4	14 295	33.30	8888	20.70	4694	10.93	15 054	35.07
	**≥ 50**	**301 9 53**	**151 340**	**50.12**	**210.8**	**74 281**	**49.08**	**39 760**	**26.27**	**14 513**	**9.59**	**22 786**	**15.06**
Western Cape (*n* = 402 537)													
	< 50	341 837	217 144	63.52	336.1	182 378	83.99	24 259	11.17	3753	1.73	6754	3.11
	50–199	86 581	41 955	48.46	281.6	27 041	64.45	9849	23.48	1939	4.62	3126	7.45
	200–1000	22 129	13 337	60.27	195.3	6707	50.29	2961	22.20	1416	10.62	2253	16.89
	> 1000	55 315	34 440	62.26	167.3	14 963	43.45	5938	17.24	2514	7.30	11 025	32.01
	**≥ 50**	**164 025**	**89 732**	**54.71**	**224.9**	**48 711**	**54.28**	**18 748**	**20.89**	**5869**	**6.54**	**6 404**	**18.28**

Note: values in bold indicate number of patients that are unsuppressed.

F/U, follow-up; VL, viral load.

## Discussion

With the recent expansion of the national ART programme and increased HIV VL testing, concerns have arisen about the impact of pre-analytical factors on VL results.^1,8^ This study explored additional factors including analytical and population-level programme dynamics, and how all these factors may play a collective role in VL result interpretation.

### Pre-analytical factors

Operational disruptions, specimen backlogs, and longer TATs were evident in Limpopo from early 2024. While the cyberattack later that year most likely worsened these issues, it probably did not affect VL results directly in subsequent months. Although many specimens were lost during June 2024 and July 2024, those processed showed no major shifts in VL distribution (Figure 4 and Figure 5). A notable change in Limpopo’s VL profile emerged only after the laboratory information system was restored in August 2024, characterised by a decline in LDL results and a rise in VLs < 50 copies/mL (Figure 5). Given the stability of other VL categories, this may reflect specimen degradation from factors such as viral particle leakage, causing LDL samples to be misclassified as < 50 copies/mL. However, previous studies have shown that HIV-1 RNA remains stable at temperatures up to 37 °C and during transit times of up to seven days, reliably differentiating suppression, LLV, and virological failure.^8,9,10,11^

In resource-limited settings, testing is usually confined to reference laboratories distant from sampling sites. For example, a Northern Cape study highlighted a 250 km radius between NHLS district laboratories, with centralised CD4 testing limited to three facilities.^12^ Such distances represent significant pre-analytical challenges that affect access and TATs.^13,14^ Although increased TATs were suspected to affect VL distribution, including for Limpopo specimens processed at GSH, no consistent correlation was found between prolonged TAT and rising LLV results (Figure 5, Online Supplementary Figure 7, and Online Supplementary Figure 8).

### Analytical factors

In light of increased reactive negative controls at GSH laboratory in November 2024 and December 2024, machine contamination was initially considered as a possible explanation for the rise in LLV results (Online Supplementary Table 6). However, when Limpopo specimen results were excluded from the dataset for GSH, the HIV VL distribution remained largely consistent with previous months. A separate analysis comparing results of Limpopo specimens processed at either GSH or Limpopo during the same time period revealed similar distribution patterns unique to the Limpopo province, regardless of the testing site.

According to the Alinity m HIV-1 assay package insert,^6^ several integrated mechanisms minimise the risk of contamination during testing. These include the use of aerosol-barrier pipette tips, which are disposed after each use to prevent cross-sample carryover, and the performance of amplification and detection within sealed reaction vessels, which are discarded following analysis. The assay also employs deoxyuridine triphosphate in place of deoxythymidine triphosphate during DNA synthesis, enabling the enzymatic degradation of any carryover amplicons by uracil-DNA glycosylase prior to the next amplification cycle. The heat-labile uracil-DNA glycosylase enzyme is inactivated during the RT-PCR process, preventing interference with the generation of new double-stranded DNA products.^15^ The carryover rate for the Alinity m HIV-1 assay was evaluated in the package insert by alternating high positive (1 000 000 copies/mL) and HIV-1-negative plasma samples across multiple runs. Of 720 negative replicates, one tested positive, yielding an overall carryover rate of 0.1% (95% confidence interval: 0.0% – 0.8%). This might explain why, despite having contamination issues during this period, the HIV VL proportions remained mostly unaffected during November 2024 and December 2024. For reference purposes, an unrelated contamination event, which occurred at GSH laboratory in 2025, where leakage of amplicons from an uncapped reaction vessel led to a significant decline in the LDL VL results, is mentioned in Online Supplementary Text 1.

Up to this point, our findings indicate that the rise in LLV results observed at GSH was driven primarily by an increase in referred specimens from Limpopo, which exhibited a distinct HIV viral load distribution compared to specimens from the laboratory’s usual catchment areas. This provincial variation in HIV VL distribution appears to be specific to region and did not correlate with pre-analytical factors such as extended transit times or compromised sample integrity as assumed previously. To explore potential reasons for these inter-provincial differences, the study was extended to examine broader population-level programme indicators, including HIV prevalence, ART coverage and adherence to treatment.

### Population-level programme factors

Comparing Limpopo and Western Cape provinces reveals marked differences in VL distribution, despite similar HIV prevalence and ART coverage (about 72% – 74%). In Limpopo, about 20% of VL results fall within the LLV ranges (50–200 copies/mL and 200–1000 copies/mL), with 70% suppressed below 50 copies/mL (including LDL). In contrast, Western Cape reports about 13% LLV and 81% suppression below 50 copies/mL (including LDL). Table 1 highlights that higher initial VL predicts a lower likelihood of suppression on follow-up, consistent with studies linking persistent LLV to virological failure and drug resistance.^16,17,18,19^ These findings raise questions about the adequacy of the current virological failure threshold of > 1000 copies/mL, which was established by the World Health Organization for dried blood spot testing in decentralised settings.^20^ Lowering it to 400 copies/mL could allow earlier detection of treatment failure, reduce transmission risk and accommodate for viral blips, while enabling resistance testing when needed.

Follow-up rates among unsuppressed patients are suboptimal in both provinces—50.12% in Limpopo and 54.71% in Western Cape. Interestingly, the VL > 1000 category shows the highest follow-up rate and the shortest time to follow-up, yet it also had the lowest rate of virological suppression. Observing the general trend, Western Cape province has higher follow-up and suppression rates, alongside lower LLV proportions on follow-up (Table 1), suggesting that adherence challenges can, at least in part, explain the differences observed in VL result distributions between the two provinces. Barriers to ART adherence are multifactorial, including side effects, nondisclosure, and stigma.^21,22^ Structural and socioeconomic factors, such as limited healthcare access and long travel distances, are especially severe in provinces such as Limpopo. These systemic inequities affect rural and under-resourced communities disproportionately, necessitating targeted interventions.

### Limitations

Information regarding disruptions to services, such as ART access, or other epidemiological factors that could have influenced HIV VL results during the study period is uncertain. The exact pre-analytical handling of specimens in Limpopo is also unclear, including whether samples were centrifuged upon arrival or only just prior to processing on the Alinity m platform. In addition, details about specimen transport conditions are lacking, particularly as temperature loggers were not used.

Regarding the Python-based record linkage script, the records were first pre-clustered according to the first letter of the patient surname before any pairwise comparisons were done to match similar records. As records in different clusters are never compared against each other, patients with typographical errors in the first letter of their surname, or use of a different surname (e.g., married women) or instances where name and surname are switched around were not linked to each other and were treated as separate patients. Other relevant patient clinical information, such as treatment interruption, was not available for this study.

### Conclusion

This study highlights the value of routine data analysis in high-throughput laboratory settings, where HIV VL results are frequently auto-released without direct oversight by a pathologist. Aggregating VL data helps to identify distribution trends, which tend to remain relatively consistent over time. Establishing a baseline for these patterns allows for the early identification of anomalies and reduces the risk of inaccurate results being reported unintentionally.

Differences in HIV VL distribution between provinces appear to be driven more by epidemiological factors, such as ART adherence, than by laboratory-related pre-analytical variables. Even in real-world conditions where TATs reached up to 10 days, no significant effect was observed on VL outcomes (Figure 5). Similarly, the cyberattack on the information technology system did not appear to alter HIV VL result distributions, although it seemed to have a chain effect later on, disrupting normal laboratory operations and referral pathways, leading to specimens being rerouted to external reference laboratories outside the province.

Large volumes of specimens sent to GSH from Limpopo owing to local backlogs still showed a distinct and consistent VL distribution pattern, different from that of the Western Cape, regardless of transit duration or test location (Figure 6). This event highlights the potential for future multi-centre verification and validation, particularly in cases where significant and unexplained deviations in HIV VL results occur together with other confounding factors like contamination, which can further complicate the interpretation of results.

To understand better the broader factors influencing VL distribution, further research is needed, specifically exploring population-level HIV prevalence, ART coverage, and adherence. One major obstacle is the lack of a consistent unique identifier or medical record number across provinces; currently, only the Western Cape captures these data systematically. Analyses like this can provide objective insights into ART adherence and can highlight areas where policy changes — such as expanding access to VL testing, including point-of-care options^23^ — could help to improve adherence and move closer to achieving the remaining targets of the Joint United Nations Programme on HIV/AIDS 95–95–95 strategy.
